# Clinical and cost-effectiveness of physiotherapy interventions following total hip replacement: a systematic review and meta-analysis

**DOI:** 10.1007/s00296-020-04597-2

**Published:** 2020-05-25

**Authors:** Francis Fatoye, J. M. Wright, G. Yeowell, T. Gebrye

**Affiliations:** Department of Health Professions, Faculty of Health, Psychology, and Social Care, Manchester Metropolitan University, Brooks Building, 53 Bonsall Street, Manchester, M15 6GX UK

**Keywords:** Cost-effectiveness, Physiotherapy, Total hip replacement, Systematic review

## Abstract

To examine the reported clinical and cost-effectiveness of physiotherapy interventions following total hip replacement (THR). A systematic review was completed according to the Preferred Reporting Items for Systematic Reviews and Meta-Analyses (PRISMA). MEDLINE, CINAHL, AMED, Scopus, DARE, HTA, and NHS EED databases were searched for studies on clinical and cost-effectiveness of physiotherapy in adults with THR published up to March 2020. Studies meeting the inclusion criteria were identified and key data were extracted. Risk of bias was assessed using the Cochrane Risk of Bias Tool and a Consolidated Health Economic Evaluation Reporting Standards (CHEERS). Data were summarised and combined using random-effect meta-analysis. A total of 1263 studies related to the aim of the review were identified, from which 20 studies met the inclusion criteria and were included in the review. These studies were conducted in Australia (*n* = 3), Brazil (*n* = 1), United States of America (USA) (*n* = 2), France (*n* = 2), Italy (*n* = 2), Germany (*n* = 3), Ireland (*n* = 1), Norway (*n* = 2), Canada (*n* = 1), Japan (*n* = 1), Denmark (*n* = 1), and United Kingdom (UK) (*n* = 1). The duration of follow-up of the included studies was ranged from 2 weeks to 12 months. Physiotherapy interventions were found to be clinically effective for functional performance, hip muscle strength, pain, and range of motion flexion. From the National Health Service perspective, an accelerated physiotherapy programme following THR was cost-effective. The findings of the review suggest that physiotherapy interventions were clinically effective for people with THR. However, questions remain on the pooled cost-effectiveness of physiotherapy interventions, and further research is required to examine this in patients with THR. Future studies are required to examine the cost-effectiveness of these interventions from patients, caregivers, and societal perspectives.

*Registration Prospero* (ID: CRD42018096524).

## Introduction

Osteoarthritis (OA) is one of the major chronic diseases, and a primary cause of pain and disability among adults [1, 2]. Hip and knee OA ranked as the 11th highest contributor to global disability and 38th highest in disability-adjusted life years (DALYs) [3]. Between 1990 and 2010, the global age-standardised prevalence of hip OA was 0.85% [95% uncertainty interval (UI) 0.74–1.02%]. For people age ≥ 60 years, the prevalence of radiographic hip OA (7%) is less common than OA of the knee (37%) [4]. The prevalence of OA of the hip is higher in females than males [3]. Due to the severe long-term pain and disability resulting from OA hip, its clinical and economic impact is substantial. People with OA of the hip have difficulty with functional activities as well as high levels of depression and anxiety [5, 6]. The total costs of OA in the United States of America (USA), France, United Kingdom (UK), Canada, and Australia accounted for between 1 and 2.5% of the Gross National Product (GNP) for these countries [7]. In contrast, the cost of OA in Hong Kong accounted for 0.28% of the GNP which was between £253 million and £308 million [7]. From this, the annual direct and indirect costs per person ranged from £384 to £883 and £261 to £525, respectively [8].

Pharmaceutical management, non-pharmaceutical therapies, and surgical procedures are advocated by clinical guidelines for managing OA of the hip [9]. Total hip replacement (THR) is a common orthopaedic procedure for OA of the hip when conservative management fails [10]. Evidence showed that around 2.5 million (1.4 million women and 1.1 million men) Americans are living with a THR [11]. Current clinical guidelines recommend that non-pharmaceutical therapies including access to appropriate information to enhance understanding of the condition; activity and exercise; positive behavioural changes; manipulation and stretching; and transcutaneous electrical nerve simulation for patients following a THR for hip OA [12].

Previous systematic reviews have evaluated the effectiveness of physiotherapy interventions following THR; however, they reported conflicting findings [13, 14]. Lowe et al. [13] indicated that physiotherapy exercise following THR has the potential to benefit patients. On the other hand, Wijnen et al. [14] identified that there was limited evidence to support the effectiveness of physiotherapy exercise following THR. Furthermore, there are no reviews that have been conducted on the cost-effectiveness of physiotherapy interventions following THR. Therefore, the purpose of this review was to investigate the clinical and cost-effectiveness of physiotherapy interventions following THR, which could be used to inform clinical practice and patient decision-making.

## Methods

### Search protocol and registration

This systematic review used the Preferred Reporting Items for Systematic Reviews and Meta-Analysis (PRISMA), a technique that addresses the eligibility, data sources, selection of studies, data extraction, and data analysis as a reporting guideline [15]. This review was registered on PROSPERO, with registration number, CRD: CRD42018096524.

### Data sources

A search of literature for published and unpublished studies was conducted to MEDLINE, Cumulative Index to Nursing and Allied Health Literature (CINAHL), AMED, Scopus, Database of Abstracts of Reviews of Effects (DARE), Health Technology Assessment (HTA) database, and the National Health Service Economic Evaluation Database (NHS EED) in the last 2 decades. The search terms used were hip, replace*, “total hip replacement’’, arthroplasty, “total hip arthroplasty’’, “therapeutic exercise’’, training, “functional training’’, “home physical training’’, “joint mobilization’’, exercise, physical therapist, therap*, treatment, medicine, muscle*, quadriceps*, strength, function, kinesiotherap*, rehabilitation, physiotherapy, “exercise therapy’’, “physical therapy’’, effectiveness, “clinical effectiveness’’, cost, value, money, expenditure, QALY, HRQoL, “healthcare costs’’, economics, “cost-effectiveness analysis’’, “cost-utility analysis’’, and “cost–benefit analysis’’. These search terms were combined using conjunctions such as “AND’’ and “OR’’.

### Search strategy

The Population, Intervention, Comparison, Outcome (PICO) framework was utilized in the development of the search strategy with search terms and limits relating to population of interest and intervention. The inclusion criteria were studies that: included patients (mean age ≥ 18 years) following THR for hip OA; assessed the clinical or cost-effectiveness of different forms of physiotherapy compared to other forms of physiotherapy or no intervention; reporting results of randomized-controlled and retrospective/prospective trials. In this review, physiotherapy interventions covered a range of techniques including massage, passive stretching, functional rehabilitation, interdisciplinary rehabilitation, exercise, physical training, acupuncture, spinal manipulation, advice, yoga, cognitive behavioural therapy, and martial arts. The economic evaluation (cost-effectiveness analysis, cost–benefit analysis, and cost–utility analysis) carried out alongside randomized-controlled trials and retrospective cohort study were included.

The outcomes of interest in this review included: pain, function, muscle strength, clinical and motor performance, activities of daily living, and health-related quality of life. To be included for the economic evaluation, studies had to relate the costs of the interventions to the effects of the interventions. Systematic reviews, narrative literature reviews, studies of non-English language, and conference papers were excluded. Further exclusion criteria were abstract unavailable, studies not yet fully completed, and studies carried out with THR patients mean aged < 18 years.

Duplicates were removed electronically and manually. Two independent researchers (TG and FF) were involved in screening the title and abstract of each study. Full-text articles were obtained and were excluded if they did not meet the inclusion criteria. Any disagreement in study selection was resolved through discussion and consultation with other members of the team (GY and JMW) where necessary.

### Data extraction and risk of bias assessment

One of the researchers extracted data (TG) and the three members of the team cross-checked the extracted data (FF, GY, and JMW). The following data were extracted: author and date of the study, the location/country, type of participant, and the number of participants involved in the study. The mean age, percentage of male and female participants who received the interventions and the control arm, and the type and the duration of the physiotherapy interventions were also extracted from each study. Furthermore, data regarding outcome measures, including the primary and secondary health outcomes, resource use and cost, and the cost-effectiveness ratio (ICER) were extracted.

Risk of bias for studies that met the inclusion criteria for the clinical effectiveness was assessed using the criteria of the Cochrane Risk of Bias Tool [16]. The Cochrane Collaboration’s tool aims to make the process clearer and more accurate, and it covers six domains of bias such as selection bias, performance bias, detection bias, attrition bias, reporting bias, and other bias. Studies were considered high risk of bias when one or more of the key domains had unclear or high risk of bias [16].

The Consolidated Health Economic Evaluation Reporting Standards (CHEERS) statement was also used as a reporting guideline for the included cost-effectiveness studies [17]. Twenty-four items were addressed in six categories, which include title and abstract, introduction, methods, results, discussion, and others. Cost-effectiveness studies were rated positive (√) if they reported in full, and negative (*x*) if they did not fulfil the listed criteria in the CHEERS statement. For those studies that have partial or inconclusive information, they were labelled as partial (*P*). A total score of 1 was assigned if they fulfilled the requirement of reporting for that Item completely, 0 for not reporting and 0.5 for partial reporting. The maximum score for an article that reported completely all information was 24.

### Data analysis

A descriptive synthesis and meta-analysis of the extracted data is presented. This study considered a weighting procedure for the clinical effectiveness of physiotherapy interventions as well as its cost-effectiveness of the included studies only when the procedure for combining data from multiple studies was satisfied. The continuous outcomes measures were expressed as a weighted mean difference with 95% confidence intervals. To summarise the findings across the studies, a statistical significance of *p* < 0.05 was set. Due to the statistical evidence of heterogeneity across the studies, a random-effects model was chosen [15].

## Results

**Fig. 1:**
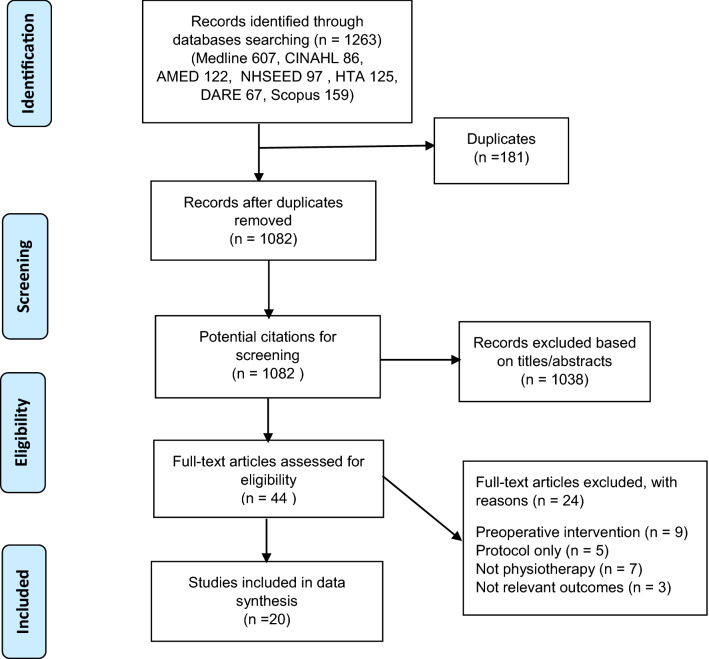
Systematic review flow diagram

From the literature search, 1263 potentially relevant studies were identified. Of these, 181 duplicates were removed. The title and abstract of the remaining 1082 studies were screened for eligibility. The full texts of 44 remaining studies were reviewed. Overall, 20 studies were eligible and included in this review. A summary is provided in the systematic review flow diagram (Fig. 1).

### Study characteristics

Eighteen studies assessed the clinical effectiveness of physiotherapy interventions and two studies examined cost-effectiveness of the interventions using information from 1400 and 108 patients following THR, respectively. The duration of follow-up of patients in the included studies ranged from 2 weeks to 12 months. The mean age of the participants in the intervention and control groups ranged from 46.93–68.6 years and 55.5–68.58 years, respectively. The geographical locations of these studies were: Australia (*n* = 3), Brazil (*n* = 1), USA (*n* = 2), France (*n* = 2), Italy (*n* = 2), Germany (*n* = 3), Ireland (*n* = 1), Norway (*n* = 2), Canada (*n* = 1), Japan (*n* = 1), Denmark (*n* = 1), and UK (*n* = 1) (Table 1).

**Table 1:** Summary of the characteristics of the studies reporting the clinical effectiveness

Reference/country/duration	Participants			Interventions	Control	Effectiveness
	Number	Mean (SD) age	% of female			
Umpierres et al. [31]/Brazil/2 weeks	Total = 106 Int = 54; Cot = 52	Total = 61.4 (15.0) Int = 61.8 (15.6); Cot = 60.9 (14.5)	Int = 51.9 Cot = 55.8	Verbal instructions and demonstrations associated with daily exercise practice guided by a physiotherapist	Verbal instructions and physiotherapy exercise demonstrations	Flexion: Int = 4.3 (0.1); *p* = < 0.001 Cot = 3.9 (0.7); *p* = < 0.001 MD = 0.807 [0.411, 1.204, *p* = 0.00] Extension: Int = 4.5 (0.1); *p* = 0.004 Cot = 4.1 (3.1); *p* = < 0.001 MD = 0.184 [− 0.197,0.566, *p* = 0.34] Motor performance, Int = 8.6 (0.1); *p* = 0.03 Cot = 8.3 (0.1), *p* = 0.16 MD = 3 [2.44, 3.55, *p* = 0.00] Clinical (pain), Int = 4.1 (0.1); Cot = 3.4 (0.1)
Haas et al. [19]/Australia/1 year	Total = 276 Int = 130; Cot = 146	Int = 67.77 (10.62) Cot = 68.58	Int = 58 Cot = 62	Acute weekend physiotherapy service	No physiotherapy	Int: Utility (Median, IQR) = 0.54 (0.31, 0.67) Pain (median, IQR) = 6 (5, 7); Cot: Utility = 0.55 (0.30, 0.70); Pai* n* = 5 (5, 7)
Naylor et al. [24]/Australia/1 year	Total = 246 Int = 123; Cot = 123	Int = 67.8 (10) Cot = 66.9 (10.6)	Int = 36.8 Cot = 31.7	Inpatient physiotherapy	No physiotherapy	Int: (3 months, 1 year): Oxford Hip Score (OHS) median (IQR) = 46 (41, 48); 48 (46 48) *p* = 0.6; EuroQol scale, Median (IQR) = 85 (80, 95); 85 (75, 95) *p* = 0.09 Cot: (3 months, 1 year): Oxford Hip Score: 46 (41, 48); 48 (46, 48); EuroQol = 90 (80 95); 90 (80, 95)
Trudelle-Jackson and Smith [36]/USA/8 weeks	Total = 34 Int = 18; Cot = 16	59.5 (11.2)	N/A	Sitting: sit to stand. Standing: unilateral heel raises, partial knee flexion, single leg stand, knee raises with alternate arm raises, side and back leg raises, unilateral pelvic raising, and lowering. Repetition rate (RR) = 15, 3 to 4 × week for 8 weeks. If able RR increased to 20 at 1st follow-up (2 weeks) and 2 × 20 at 2nd follow-up (8 weeks)	Gluteal muscle sets, hamstrings and quadriceps sets, ankle pumps, heel slides Hip abduction in supine, internal rotation, and external rotation. RR as for intervention group	Flexors Int: Hip Questionnaire-12 (median, range) = 16 (12, 38) Cot: Hip Questionnaire-12 (median, range) = 17.5 (12, 33)
Jan et al. [20]/USA/12 weeks	Total = 53 Int = 26 Cot = 27	Int = 58.8 (12.9) Cot = 57.0 (12.8)	Int = 34 Cot = 37	Patients underwent a 12-week home program that included hip flexion, range of motion exercises for both hip joints; strengthening exercises for bilateral hip flexors, extensors, and abductors; and a 30-min walk every day	No training	Flexors, Int = 57.5 (22.3); Cont = 50.8 (21.2) MD = 0.31 [− 0.23, 0.85, *p* = 0.26] Function score, Int = 13.1 (0.6); *p* < 0.05 Cot = 12.0 (1.4), MD = 0.922 [0.356, 1.49, *p* = 0.001]
Husby et al. [37]/Norway/5 weeks	Total = 24 Int = 12 Cot = 12	Int = 58 (5) Cot = 56 (8)	Int = 58 Cot = 66	Patients performed maximal strength training (STG) in leg press and abduction with the operated leg only five times a week for 4 weeks in addition to the conventional rehabilitation program	Patients received supervised physical therapy three-to-five times a week for 4 weeks	1-repetition maximum increased in the bilateral leg press (*p* < 0.002) and in the operated leg separately (*p* < 0.002) in the intervention compared with the control
Monaghan et al. [26]/Ireland/18 weeks	Total = 63 Int = 32 Cot = 31	Int = 68(8); Cot = 69 (9)	Int = 37 Cot = 26	The participants were taught 12 exercises by the supervising physiotherapist. They also attended classes twice weekly for 6 weeks, and were not given any additional exercises as a home exercise program	All patients were advised to walk daily with crutches until review by the orthopaedic surgeon at 6 weeks, increasing the distance gradually to approximately 1 mile after 1 month	Mean % at week 18 (Int vs Cot) WOMAC (pain) = − 0.81 (− 1.8 to 0.2), *p* = 0.1; WOMAC (stiffness) = − 0.44 (− 1.2 to − 0.28); *p* = 0.2; WOMAC functio* n* = − 4.0 (− 0.71 to 1.0); *p* = 0.04
Winther et al. [35]/Norway/12 months	Total = 63 Int = 31 Cot = 29	Int = 61 Cot = 66	Int = 54 Cot = 52	Patients were trained at 85–90% of their maximal capacity in leg press and abduction of the operated leg (4 × 5 repetitions), 3 times a week at a municipal physiotherapy institute up to 3 months postoperatively	Patients were followed a training program designed by their respective physiotherapist, mainly exercises performed with low or no external loads.	Int. patients were substantially stronger in leg press and abduction than Cot
Okoro et al. [18]/UK/6 weeks	Total = 49 Int = 25 Cot = 24	Int = 65.15 (9.06) Cot = 66.3 (11.02)	Int = 15/25, Cot = 10/24	Patients were instructed to perform a range of repetitions (0–3, 4–6, 7–10) depending on their initial physiotherapy assessment and then to progress, when able to, to achieve progressive overload. Subjects were encouraged to exercise at least 5 times a week	Home-based functional non-progressive resistance training exercises that were geared towards getting the patients safely mobile	Maximal voluntary contraction of the operated leg quadriceps (MVCOLQ); MD = 26.50 (8.71) *p* = 0.001; timed up and go (TUG); MD = − 1.44 (0.45); *p* = 0.0001 Stair Climb Performance (SCP); MD = − 3.41(0.80); *p* = 0.0001 6 min Walk Test (6MWT); MD = 45.61 (6.10)m; *p* = 0.0001
Maire et al. [27]/France/6 weeks	Total = 14 Int = 7 Cot = 7	N/A	N/A	Muscular strength, range of motion, aquatics, walking 2 h/day). In addition, this group undertook an arm-interval exercise program with an arm ergometer	Muscular strength, range of motion, aquatics, walking 2 h/day	Int: WOMAC (pain) = − 100; *p* < 0.05; WOMAC (physical function) = − 45; *p* < 0.05 Cot: WOMAC (pain) = − 72; *p* < 0.05 WOMAC (physical function) = − 26 *p* < 0.05
Beaupre et al. [32]/Canada/12 months	Total = 21 Int = 11 Cot = 10	Int = 51.7 (8.3) Cot = 55.9 (9.9)	Int = 64% Cot = 30%	Received out-patient rehabilitation program. Sessions were approximately two and one half hours in durations and included both aquatic and land-based components with a focus on strength and gait retraining	Usual care	Mean % from 6 weeks to 4 months postoperative Int: hip flexion (SD) = 73.8 (50.1) *p* = 0.69; hip extension (SD) = 50.5 (26.1); *p* = 0.78; Cot: hip flexion (SD) = 39.8 (64.1), *p* = 0.69; hip extension (SD) = 30.5 (67.3), *p* = 0.78
Nankaku et al. [33]/Japan/4 weeks	Total = 28 Int = 14 Cot = 14	Int = 60.5(6.4) Cot = 60.8 (7.5)	Int = 50 Cot = 50	Exercise program of hip external rotator was performed and supervised by an experienced physical therapist.	Usual care	Int, hip pain; *p* = 0.05; hip flexion angle, *p* = 0.05; hip abduction angle, *p* = 0.05 Cot, hip pain; *p* = 0.05; hip flexion angle, *p* = 0.05; hip abduction angle, *p* = 0.05
Beck et al. [21]/Germany/12 months	Total = 160 Int = 80 Cot = 80	Int = 59^®^ Cot = 61.9^®^	Int = 52.5 Cot = 63.8	Intensive exercise therapy: walking slowly in circles, fast walking, leg axis training from various start positions, correct sitting, and team circles games	No exercise therapy	Int WOMAC (pain) = 100, Cot = 95; *p* = 0.003 Int EQ-5D (VAS) = 90; Cot = 85; *p* = 1.00 Int WOMAC (stiffness) = 87.5; Cot = 100; *p* = 0.373
Maire et al. [28] /France/12 months	Total = 14 Int = 7 Cot = 7	N/A	N/A	Muscular strength, range of motion, aquatics, walking 2 h/day). In addition, this group undertook an arm-interval exercise program with an arm ergometer	Muscular strength, range of motion, aquatics, walking, 2 h/day	Int: WOMAC (physical function) = 5 (3–15); *p* < 0.05; Walking distance (m) = 486 (343–584) Cot: WOMAC (physical function) = 14 (4–18); walking distance = 398 (333–482)
Galea et al. [25]/Australia/8 weeks	Total = 23 Int = 11 Cot = 12	Int = 68.6 (9.7) Cot = 66.6 (7.9)	Int = 8/11 Cot = 8/12	Advice about how to progress the exercises. The maximum time period for each exercise was 5 min, which included a rest period if required	Patients were not given any further instruction on progressing or modifying the exercises	Int: WOMAC (pain), *p* = 0.07; stiffness, *p* = 0.26; quality of life, 0.02; Cot: WOMAC (pain), *p* = 0.08; stiffness *p* = 0.34; quality of life, *p* = 0.02
Giaquinto et al. [29]/Italy/6 months	Total = 64 Int = 31 Cot = 33	Int = 70.6 (8.4); Cot = 70.1 (8.5)	Int = 66.6 Cot = 67.7	The hydrotherapy group was treated in a special pool for 40 min after 20 min of passive joint motion, during which participants were prepared	Patients received land therapy followed by a ‘neutral’ massage on the hip scar for 20 min	Int: WOMAC (pain), *p* < 0.01; WOMAC (stiffness), *p* < 0.01; WOMAC (function) *p* < 0.01 Cot: WOMAC (pain), *p* = 0.08; stiffness, *p* = 0.58; function, *p* = 0.01
Monticone et al. [30]/Italy/12 months	Total = 100 Int = 50 Cot = 50	Int = 69.5 (7.5); Cot = 68.8 (8.1)	Int = 32/50 Cot = 28/50	Performed task-oriented exercises, such as moving from a sitting to a standing position, etc. Sessions of stationary cycling were added to optimise hip strength and mobility	Performed open kinetic chain exercises	WOMAC (function), *p* < 0.001; WOMAC (pain) *p* < 0.001; WOMAC (stiffness) *p* < 0.001
Mikkelsen et al. [34]/Denmark/10 weeks	Total = 62 Int = 32 Cot = 30	Int = 64.8 (8); Cot = 65.1 (10)	Int = 44 Cot = 40	Patients warmed up on a stationary bike for 5–10 min and then performed unilateral patient resistance training of the operated leg for 30–40 min. One-to-one supervision by physiotherapists	Patients were recommended to perform one set of ten repetitions twice a day in their maximum possible range of motion	Ten weeks, maximum walking speed Int = 11.08, Cot = 11.99, *p* = 0.008; hip abduction strength, Int = 1.03 (0.3), Cot = 1.03 (0.3); *p* = 0.26; hip flexion strength, Int = 1.25 (0.3); Cot = 1.32 (0.4); *p* = 0.29

*Int* intervention, *Cot* control, *MD* standard mean difference, *USA* United States of America, % percentage, *WOMAC* Western Ontario and McMaster Universities (WOMAC) Osteoarthritis Index, ^®^ Median

### Risk of bias

The assessment of risk of bias of the included clinical effectiveness studies is presented in Table 2. All the included studies have unclear or high risk of bias within at least one domain, and thus, no studies have achieved a low risk of bias. Except two studies that were assigned high risk of bias [18] and unclear risk of bias [19] for reporting bias, most of the included studies achieved low risk of bias for the reporting and other bias. Thirteen and 16 out of 18 studies had high risk of bias for treatment allocation and blinding of participants of intervention. Sixteen out of eighteen studies had low risk of bias and two studies [20, 21] had unclear risk of bias for blinding outcome assessment. Five of eighteen studies were assigned unclear attrition bias, and the remaining studies had low risk of bias.

**Table 2:** Summary of risk of bias assessment

	Random sequence generation (selection bias)	Allocation concealment (selection bias)	Blinding of participants and personnel (performance bias)	Blinding of outcome assessment (detection bias)	Incomplete outcome data (attrition bias)	Selective reporting (reporting bias)	Other bias
Umpierres et al. [31]	+ 1	+ 1	− 1	+ 1	+ 1	+ 1	+ 1
Haas et al. [19]	− 1	− 1	− 1	+ 1	+ 1	?	+ 1
Naylor et al. [24]	− 1	− 1	− 1	+ 1	+ 1	+ 1	+ 1
Trudelle-Jackson and Smith [36]	?	− 1	1	+ 1	?	+ 1	+ 1
Jan et al. [20]	− 1	− 1	− 1	?	+ 1	+ 1	+ 1
Husby et al. [37]	+ 1	− 1	− 1	+ 1	?	+ 1	+ 1
Monaghan et al. [26]	+ 1	+ 1	− 1	+ 1	+ 1	+ 1	+ 1
Okoro et al. [18]	+ 1	+ 1	−	+ 1	?	− 1	+ 1
Maire et al. [27]	?	− 1	− 1	+ 1	+ 1	+ 1	+ 1
Beaupre et al. [32]	+ 1	− 1	1	+ 1	+ 1	+ 1	+ 1
Nankaku et al. [33]	+ 1	− 1	− 1	+ 1	+ 1	+ 1	+ 1
Maire et al. [28]	+ 1	− 1	− 1	+ 1	+ 1	+ 1	+ 1
Galea et al. [25]	+ 1	− 1	− 1	+ 1	+ 1	+ 1	+ 1
Giaquinto et al. [29]	?	− 1	− 1	+ 1	?	+ 1	+ 1
Monticone et al. [30]	+ 1	+ 1	− 1	+ 1	+ 1	+ 1	+ 1
Mikkelsen et al. [34]	+ 1	+ 1	− 1	+ 1	+ 1	+ 1	+ 1
Winther et al. [35]	?	− 1	− 1	?	+ 1	+ 1	+ 1
Beck et al. [21]	?	− 1	− 1	+ 1	?	+ 1	+ 1

+ 1, low risks of bias, **−** 1, high risk of bias, ?, unclear risk of bias

In relation to the two included cost-effectiveness studies [22, 23], the CHEERS scores suggest that the methodological quality of the included studies had adequate quality (Table 4).

### Effectiveness of physiotherapy interventions

The effectiveness of physiotherapy interventions was assessed in the included studies.

### Acute hospital length of stay

Haas et al. [19] investigated the effect of an acute weekend physiotherapy service compared to no physiotherapy service following THR. Weekend physiotherapy service was associated with significantly increased odds of discharge directly home [odds ratio 3.151 (1.039–9.555)] and improved mobility [coefficient 4.301 (1.500–7.101)]. However, patients in the intervention group perceived hospitalisation as less helpful and acute length of stay was longer compared to patients without physiotherapy services at the weekend. Overall, weekend physiotherapy service was beneficial on discharge destination and patient mobility.

### Health-related quality of life

Three studies reported the impact of physiotherapy interventions on health-related quality of life (HRQoL) [21, 24, 25]. The Euroqol visual analogue scale [21, 24] and a self-administered HRQoL questionnaire [25] were used to assess the quality of life of THR patients. The comparative advantage of a targeted home- and centre-based exercise programme over unsupervised home-based exercise group were examined in patients following THR [25]. Patients who received the targeted home- and centre-based exercise programme achieved significant improvements (*p* < 0.05) in HRQoL. On the other hand, no clinically significant difference was observed between patients following THR in the groups who received inpatient and sports rehabilitation compared with control on HRQoL at 1 year [21, 24].

### Function

The effects of physiotherapy interventions on functional performance in patients following THR were assessed in six studies [20, 26–30]. The findings of these studies demonstrated that hydrotherapy, home exercise programme, physiotherapy-led functional exercise program, a 6-week arm exercise programme, an arm-interval exercise program, in-hospital program based on task-oriented exercises, and a targeted home and centre exercise programme were effective in improving the functional performance of patients following THR. One of the studies [20] used Harris Hip Score to measure function, whereas the remaining five studies [26–30] used Western Ontario and McMaster Universities (WOMAC) Osteoarthritis Index.

### Muscle strength

Six studies investigated the effects of physiotherapy interventions on hip flexors muscle strength following THR [26, 31–35]. Compared to patients assigned into the control, improved hip muscle strength was observed in patients following THR who received home exercise programme, postoperative exercise programme, exercise programme focussing on hip external rotator muscle, supervised progressive resistance training, rehabilitation and muscle strength training.

### Range of movement

Range of motion flexion data suitable for meta-analysis were available from two studies that compared physiotherapy and no physiotherapy interventions [20, 31]. As it is demonstrated in Table 3, there was evidence that physiotherapy interventions significantly improved range of motion flexion with a standard difference in means 0.634 (95% CI 0.170, 1.098, *p* = 0.007).

**Table 3:**
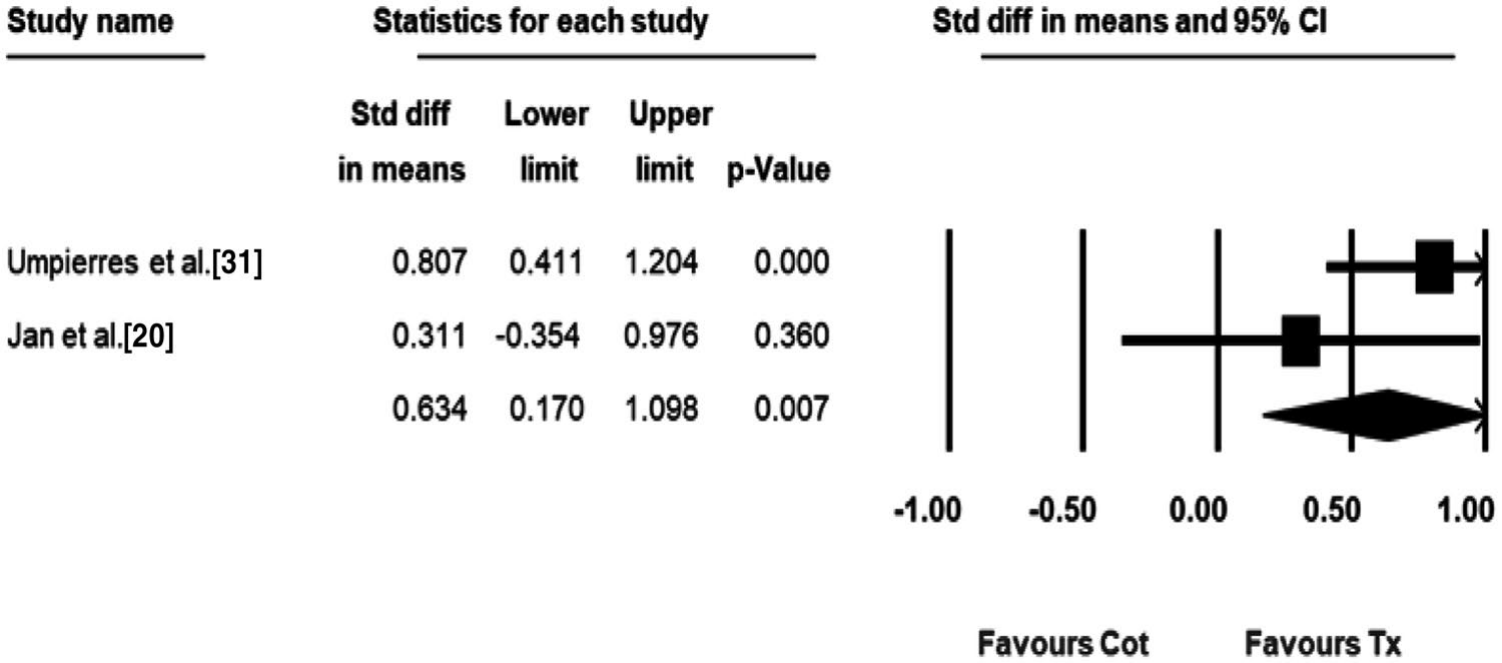
Forest plot of the mean difference I hip flexion for total hip replacement between physiotherapy and without physiotherapy

*Cot* control, *Tx* treatment, *CI* confidence interval

### Pain

The effectiveness of physiotherapy on hip pain following THR was examined in seven studies conducted across different countries [20, 26, 27, 29–31, 33]. The findings of five of the studies [27, 29–31, 33] showed that hip pain was significantly improved for those patients following THR in the intervention group compared to control. Whereas two studies [26, 31] reported that patients following THR received home- and centre-based exercise and physiotherapy supervised functional exercise programme showed no significant improvement in hip pain.

### Clinical and motor performance

One study [31] reported the effect of physiotherapy interventions on clinical and motor performance. The patients (*n* = 54) received rehabilitation assisted by the multidisciplinary hip group with the presence of physiotherapy professionals. After the 15th postoperative day after discharge, those in the intervention groups showed greater improvements in clinical (gait, pain, and mobility) and motor performance (gait and pain) (*p* < 0.001) compared with those patients supported without physiotherapy professionals. Those in the intervention group have also showed significantly greater improvements in muscle strength force (flexion, *p* < 0.001; extension, *p* < 0.001; abduction, *p* = 0.003; internal rotation, *p* < 0.001; external rotation, *p* < 0.001) compared to the non-intervention group.

### Cost-effectiveness of physiotherapy interventions

Two of the included studies that compared accelerated physiotherapy with standard physiotherapy [22] and in-patient rehabilitation with out-patient physiotherapy [23] have conducted economic evaluation in patients of OA following THR (Table 4). The design of the studies was a cost–utility analysis alongside randomized-controlled trial [22] and retrospective cohort study [23]. From the National Health Service (NHS) and healthcare insurer perspective, a £504 per patient [22] and € 9,126.00 [23] costs were estimated for the accelerated physiotherapy and in-patient rehabilitation, respectively. The incremental cost-effectiveness ratio estimate by Fusco et al. [22] and Krummenauer et al. [23] was £1,538/QALY and −€841/QALY gained, respectively. Overall, inpatient rehabilitation [23] was not cost-effective, whereas accelerated physiotherapy was associated with cost savings to the NHS of £200 per patient and additional 0.13 QALY [22].

**Table 4:** Summary of the characteristics of the studies reporting the clinical outcomes and cost-effectiveness for patients of THR

Study/location/study design/time-horizon	Population	Intervention	Control	Outcomes/measurement used	Cost/perspective	Results (Int vs Cot)	/24^φ^
Fusco et al. [22]; UK/cost-utility analysis/12 months	#80	Accelerated physiotherapy re-education to increase walking distance and direction and reduce reliance on aids	Standard physiotherapy	EuroQol EQ-5D	Direct cost/National Health Service	Cost I* n* = £504 per patient Cot = £705 per patient Effectiveness Int = 0.91 (0.03) Cot = 0.73 (0.05) Cost-effectiveness Int. was cost-effective than Cot	22
Krummenauer et al. [23] Germany/cost-effectiveness analysis/6 months	#28	In-patient physiotherapy	Out-patient physiotherapy	WOMAC score (%), utility, quality adjusted life years	Direct costs/healthcare insurer	Cost Int = €9126.00; Cot = €8706.00 Effectiveness Int = 38% before, and 87% after surgery (WOMC score) Cot = 41% before, and 88% after surgery Cost-effectiveness Cost/effect = €420 [198, 475]/0.77 [95% CI − 2.13, 3.18] QALYs = −€841/QALY (*p* = 0.791) Inpatient rehabilitation was not cost-effective compared to out-patient rehabilitation	20

*Int* intervention, *Cot* control, *WOMAC* Western Ontario and McMaster Universities (WOMAC) Osteoarthritis Index

^φ^CHERS Quality score

## Discussion

This is the first systematic review and meta-analysis on clinical and cost-effectiveness of physiotherapy interventions following THR. The search strategy identified 20 clinical and cost-effectiveness studies on physiotherapy interventions from Australia, Brazil, USA, France, Italy, Germany, Ireland, Norway, Canada, Japan, Denmark, and United Kingdom. The risk of bias in these studies was assessed using the Cochrane Risk of Bias Tool. All of the 18 studies included for the clinical effectiveness of physiotherapy interventions in the review had unclear or high risk of bias. The methodological quality of the two cost-effectiveness studies was assessed as adequate.

In line with the findings a systematic review by Lowe et al. [13] on clinical effectiveness of physiotherapy exercise following hip arthroplasty for osteoarthritis, the present study confirmed that physiotherapy interventions improved physical function, health-related quality of life, mobility, and muscle strength. In addition, the findings of our review showed that physiotherapy interventions improved self-perceived function, postural stability, fast-walking speed, stair climbing, and discharge destination following THR. On the other hand, physiotherapy interventions did not reduce hospital length of stay, fear of falling, hip pain, and function. Furthermore, compared to out-patient physiotherapy interventions, inpatient physiotherapy interventions following THR did not show a significantly superior cost-effectiveness from a healthcare insurer perspective.

The results of the meta-analysis of two studies [20, 31] also showed that physiotherapy intervention was beneficial compared to a control, which contradicts the findings of Lowe and colleagues [13]. Their review which focussed specifically on an outcome measure of range of motion such as flexion, extension defect, and abduction that combined data from four studies showed that there were no statistically significant differences between groups for hip joint range of motion. One possible reason for the contradiction may be the characteristics of the physiotherapy interventions such as exercise, duration of follow-up, and the outcome measures used in the individual studies.

We have adopted a robust search strategy to locate and identify all potential studies that investigated the effectiveness and cost-effectiveness of physiotherapy interventions including exercise, massage, taping, kinesiology, rehabilitation, joint mobilisation, and sport. Four independent reviewers have participated in the review process, and it has been possible to include all relevant literature in this study. Due to the fact that public health practitioners and policymakers are utilizing innovative and up-to-date physiotherapy guidelines, this review has focused on studies carried out in the last 2 decades. Given the small number of studies included for this review, the clinical and cost-effectiveness of physiotherapy interventions should be interpreted with caution. The present review may have been affected by language bias. Consequently, a small number of studies published in languages other than English might have been excluded and it is difficult to generalize the clinical and cost-effectiveness of physiotherapy interventions based on the findings of this review.

## Conclusion

This review indicates that following THR, patients with OA of the hip showed significant improvement in physical function, health-related quality of life, mobility, and muscle strength with physiotherapy interventions in a short term. On the other hand, physiotherapy interventions were not effective in terms of hospital length of stay, acute length of stay, fear of falling, and hip pain and function for patients following THR. In relation to the findings of the cost-effectiveness of physiotherapy interventions in this review, it is difficult to reach a conclusion as they were based on a small number of studies. In addition, outcome measures used in future studies need to include those which measure (or reflect) the wider social determinants of health; for example, the perspectives of patients, their caregivers, and other societal perspectives.
